# A Time-governed Slide Method

**Published:** 1918-10

**Authors:** 


### PATHOLOGY AND BACTERIOLOGY

---

***A Time-Governed Slide Method for the Agglutination Test on a Series of Microbic Emulsions.*** By Captain (Local Major) W. Broughton-Alcock, R. A. M. C. (S. R.). *Jour. of the Royal Army Medical Corps*, Vol. XXX., N° 4. Page 424.

This method is one which permits of the simultaneous examination of several standard emulsions of micro-organisms and of the determination of results in three or four minutes. Army bacteriologists have since 1916 used the method of placing drops of high titer sera diluted 1/100 on a microscope slide and making emulsions in such drops from cultures or colonies of bacteria to be tested; the results being observed by the naked eye. Similarly, unknown sera have been tested in series on slides against known bacteria.

It has also been noted that agglutination is greatly facilitated by rotation of the slide on which the mixture of the serum and emulsion are placed; the contained organisms come into frequent contact with one another and the homogeneity of the serum dilution is maintained by movement.

By the method of testing serum against emulsions of a number of different organisms at the same time, the specific agglutin can not only be detected, but can be distinguished from any co- or hetero-agglutin, which may also be present in the serum, and possible errors of diagnosis by single emulsion examination can thus be avoided.

The writer has endeavored since 1912 to establish the principle that the agglutin action can be measured by the time taken for agglutination to appear in fixed dilutions of serum.

When agglutination tests are to be carried out at intervals during the progress of a case and the results compared in time measurements, the serum requires to be so far diluted that agglutination will not be instantaneous in order that a measurement may be made of the time at which agglutination appears. An increase or decrease in the agglutin content of a serum can be detected by the decrease or increase of the time taken for the agglutination of the emulsion in this dilution. Interval examinations are carried out on similar lines and results are compared.

The dilution of the serum employed for an initial agglutination examination in the case of B. typhosus, B. paratyphosi A and B, V. cholerae, Micrococcus melitensis and M. paramelitensis, 1 in 10; in the case of B. dysenteriae Shiga or Flexner-Hiss, 1 in 6. Agglutination appearing within three minutes determines a positive finding; after three and within four minutes a partially positive finding; four minutes if the full time limit is to be given to the test.

During the carrying out of many comparative examinations by the established high titre methods at the temperature of the laboratory, or at 37° C or 55° C, and by the slide method, it was found that when a specific agglutinin action was determined by a high titre method a positive result was also obtained by the slide method. Moreover, bacteriologically proven cases have shown a higher percentage of positive finding by the slide method, and there have been no inhibitory zones appearing with the dense emulsions employed.

It is of interest and value to know that while employing the *dense* emulsions of practically medium-freed micro-organisms, the writer has been able to demonstrate by the method, positive agglutination with sera in all dilutions, tested, when a zone of inhibition has appeared, with a thin emulsion and the short or long methods employed.

The agglutination test, however, has not been diagnostic in every case, but its value has been inestimable, and it has indicated in only the most exceptional cases misleading or constantly negative results. Observations have been made on more than 3,000 subjects. Bacteriological findings have given the necessary confirmation of the test in the majority of cases of infection. Its general utility may be recognized by the fact that some clinicians have been enabled thereby to test their patients' sera in their own side-rooms.

The prepared emulsions are placed in rubber-corked phials for preservation.

If an emulsion of the B. dysenteriae groups in the 1 and 6 serum dilution, or an emulsion of B. typhosus, B. paratyphosus A or B, M. melitensis or V. cholerae in the 1 and 10 dilution, shows agglutination by the formation of definite flocculi within 3 minutes and within 4 minutes, the reaction is considered partially positive to the micro-organisms contained therein.

In hot and dry climates, it has been found satisfactory to give a time limit of three minutes to the method, keeping at hand a basin of water with bars across on which the slide can rest, and over which it can be manipulated. Captain Garrow, R. A. M. C., has devised a rocking chamber into which a slide is fitted when prepared for the test.

For an immediate agglutination test, which proved satisfactory during an outbreak of plague, a serum test of 1 in 6 and 1 in 10 is employed. The 1 in 6 dilution of serum was used in the initial test.

